# Effect of shenfu injection on microcirculation in shock patients: A protocol for systematic review and meta-analysis

**DOI:** 10.1097/MD.0000000000022872

**Published:** 2020-10-23

**Authors:** Xuemei Zhang, Ting Guo, Kaichen Zhang, Wenhui Guo, Xing An, Peiyang Gao

**Affiliations:** a Department of Critical Medicine; b Department of Respiratory, Hospital of Chengdu University of Traditional Chinese Medicine, Chengdu, China.

**Keywords:** microcirculation, protocol, shenfu injection, shock, systematic review

## Abstract

**Background:**

Shock is a major public health problem worldwide. At present, the morbidity and mortality of shock patients are relatively high. Vasomotor dysfunction is 1 of the key pathological aspects of shock. Shenfu injection has been widely used for the treatment of shock in China. Pharmacological studies have suggested that Shenfu injection can reduce peripheral circulation resistance and improve microcirculation. The purpose of this study is to evaluate the effect and safety of Shenfu injection on the microcirculation of patients with shock.

**Methods:**

This review summarizes and meta-analyzes randomized controlled trials of Shenfu injection for the treatment of shock.Searched the following electronic databases: PubMed, Cochrane Library, Embase, CNKI, VIP and Wanfang Data. The Cochrane risk assessment tool was used to evaluate the methodological quality of randomized controlled trials. All tests are analyzed according to the standards of the Cochrane Handbook. Review Manager 5.3, R-3.5.1 software and Grading of Recommendations Assessment, Development, and Evaluation pro GDT web solution are used for data synthesis and analysis.

**Results:**

This review focuses on the effects of Shenfu injection on the microcirculation of shock patients (blood lactic acid level, arteriovenous oxygen saturation, arteriovenous carbon dioxide partial pressure difference, sublingual microcirculation), 28-day mortality, 28-day ICU hospitalization and adverse reaction rate.

**Conclusion:**

This review provides a clear basis for evaluating the impact of Shenfu injection on the microcirculation of shock patients, as well as the effectiveness and safety of the treatment.

## 1 Introduction

Shock is a clinical condition caused by various reasons, as the result of insufficient blood perfusion in tissues, which leads to insufficient oxygen supply, and cannot meet the needs of metabolism. This imbalance causes tissue hypoxia and lactic acidosis. If not corrected immediately, it can lead to progressive cell damage, multiple organ failure, even death. Patients with shock account for about 34% of patients admitted to the intensive care unit.^[1]^ The mortality rate of septic shock is 50% to 60%, and the mortality rate of cardiogenic shock is 60% to 80%.^[2–5]^

Despite modern medicine provides oxygen supply, fluid resuscitation, inotropic drugs and vasoactive drugs, organ support therapy and other treatments, shock mortality is still uncontrollable.^[6–8]^ At present, the morbidity and mortality of shock patients are relatively high. Although modern medicine treatment measures are given actively, the curative effect needs to be improved,^[9,10]^ especially in the lack of methods in microcirculation.

The occurrence and development of shock are closely related to vascular activity, and microcirculation is the ultimate destination of the cardiovascular system.^[11]^ Shock, defined at the cellular level, is a state in which the oxygen delivered to the cells is insufficient to maintain cell activity and support organ function. The central role of microcirculation in providing oxygen to cells determines its importance in organ function. In shock, macrocirculation changes and microcirculation dysfunction are simultaneously involved in the pathophysiology of organ failure.^[12]^

A complex and comprehensive oxygen transport network involving the lungs, heart, large vascular system and capillaries can effectively transport oxygenated blood from the blood to the tissues, where the oxygen is used for cell metabolism and waste removal. The cardiovascular system circulates blood in the large arteries and veins (macrocirculation), but the microcirculation is responsible for the regulation of blood flow and the distribution of red blood cells (RBC) in the individual, so the microcirculation is necessary for normal organ perfusion and function. Therefore it is now considered an important organ of the cardiovascular system.^[13,14]^

In a state of cardiovascular damage, the improvement of systemic hemodynamic parameters caused by treatment does not necessarily lead to the simultaneous improvement of microcirculation, which is called the “loss of hemodynamic consistency” between the macrocirculation and the microcirculation. If it persists, despite the normalization of the macrocirculation, the microcirculation disorder has not been improved, and the mortality rate of the patient is still high.^[11,13,14]^

Shenfu injection comes from the Shenfu Decoction in “Jishengfang”,its main ingredients are aconite and ginseng extract,^[15]^ which has got the state food and drug administration approved (pharmaceutical production approval number: Z20043117).In recent years, the experimental and clinical studies on the treatment of shock with Shenfu injection have gradually increased, indicating that the treatment of shock with Shenfu injection has become feasible.^[16–18]^

According to reports, Shenfu injection can effectively improve the microcirculation of shocked rats. Shenfu injection can increase the number of capillary network openings, the diameter of arterioles and blood flow velocity,increase the density and ratio of perfusion vessels, and improve the microcirculation blood flow in the capillaries.^[19]^At the same time, it can increase oxygen delivery, oxygen consumption, oxygen uptake rate, improve tissue oxygen metabolism, and reduce blood lactic acid.^[20]^

The purpose of this study is to evaluate the effect and safety of Shenfu injection on the microcirculation of patients with shock.

## 2 Methods

This study has been registered in prospective register of systematic review (http://www.crd.york.ac.uk/PROSPERO), registration number: CRD42020197285. The procedure of this protocol is based on Preferred Reporting Items for Systematic Review and Meta-Analysis (PRISMA)-Protocol guidance.^[21]^

### 2.1 Database and search strategy

The following databases were searched: 3 English medical databases (Cochrane Library, PubMed and EMBASE) and 3 Chinese medical databases (China National Knowledge Infrastructure Database [CNKI], VIP Chinese Science and Technology Journal Database [VIP] and Wanfang Data). An extensive search of the database was carried out, and the search time ended on June 31, 2020. The search strategy is based on the guidance of the Cochrane Handbook. Search terms include: (‘Shen fu injection’OR ‘Shen fu’ OR ‘Shenfu’) AND (Shock OR Circulatory Failure OR Failure, Circulatory OR Circulatory Collapse OR Collapse, Circulatory OR Hypovolemic Shock OR Shock, Hypovolemic). In order to ensure the comprehensiveness of the search, all relevant publications including academic papers and conference articles have been studied. The language is limited to Chinese and English.

### 2.2 Inclusion criteria

#### 2.2.1 Types of studies

Only randomized controlled trials are included.

#### 2.2.2 Types of participants

Male or female patients of any age or race who have been diagnosed with shock have been included. The diagnostic criteria refer to the guidelines for rescue of sepsis published by the American Academy of Critical Care Medicine in 2016,^[22]^ the clinical expert consensus statement on the classification of cardiogenic shock published by SCAI in 2019,^[23]^ and we also refer to the 2007 Chinese Medical Association Critical Care Guidance on the resuscitation of hypovolemic shock published by the Society.^[24]^

#### 2.2.3 Types of intervention

The experimental group was treated with Shenfu injection, and the control group was treated with placebo or blank control. The course of treatment was not less than 3 days. Shenfu injection is only for intravenous use, oral or other usage are not allowed. Both groups can receive the same conventional modern medicine treatment. Modern medicine basic medical treatment includes oxygen therapy, fluid resuscitation, positive muscle strength and vasoactive drugs, anti-infection, cardiopulmonary function support, prevention of oliguria, renal replacement therapy and etiological treatment.

#### 2.2.4 Types of outcome measures

Changes in microcirculation after treatment, such as blood lactic acid levels, arteriovenous oxygen saturation, arteriovenous carbon dioxide partial pressure, and sublingual microcirculation were analyzed as the main results. The patient's 28-day mortality rate, 28 days of ICU hospitalization, and adverse reaction rates were secondary results.

### 2.3 Exclusion criteria

(1) The unrelated and duplicated documents have been deleted.
(2) Animal experiments, reviews, theoretical discussions, experience summaries, and case reports.
(3) The control drug in the study is not a placebo or blank control.
(4) The drugs used in the included studies were not administered intravenously.
(5) Review articles without original data.

### 2.4 Data collection and extraction

Refer to the Cochrane collaborative network system evaluator handbook:^[25]^ importing the search results into the document management software of EndNote (version:X9;Thomson ResearchSoft, Connecticut); excluding the duplicate literature using EndNoteX9 and excluding the unrelated articles by reading the title and abstract; and reading the full text and reserving clinical studies that meet the inclusion criteria. Two researchers (XZ and TG used self-developed data extraction tables to extract data independently. Disagreements encountered in the process have been resolved through discussions with another team member (KZ) to determine the final research choice.

Data extraction content includes: general information: research ID, author, title, publication status, report source, financial support; method information: design, arm number, random sequence generation, allocation hiding, blinding, incomplete result data, selective report, Sample size calculation, baseline comparability; Participant information: diagnostic criteria, inclusion criteria, exclusion criteria, settings, population, sample size, age, gender, disease course; Intervention information: intervention comparison name, dose, treatment time, follow-up; Results; and adverse events.

**Figure 1:**
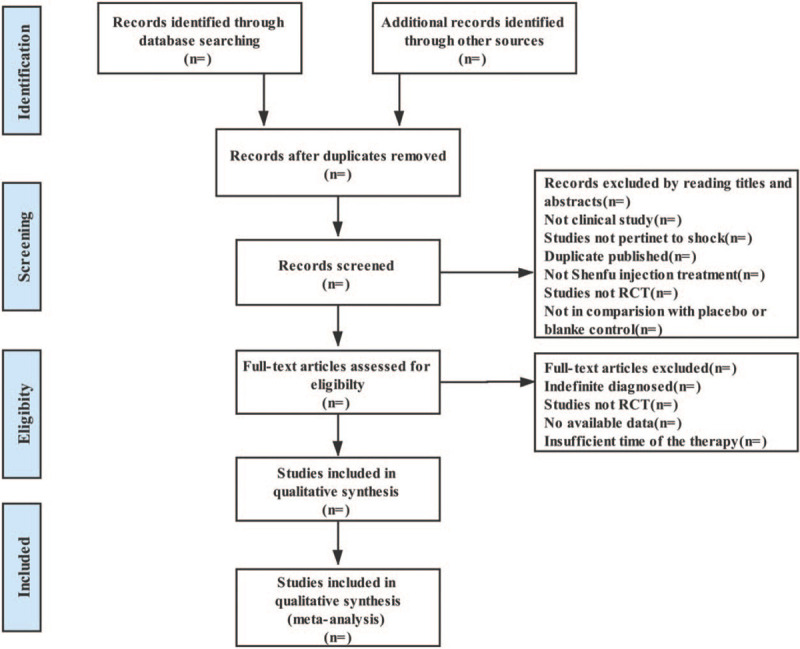
Flow chart of the selection process.^[35]^ Arrows = flow directions or reasons of the trials exclusions; RCT = randomized controlled trial.

The screening process is represented by the PRISMA flowchart:

(http://www.prisma-statement.org/) (Fig. 1).

### 2.5 Assessment of methodologic quality

The Cochrane risk assessment tool has been used.^[26]^ Deviation risk assessment is as follows: generation of allocation sequence, allocation concealment, double blind, incomplete result date, selective result report, follow-up and other deviations. These areas are classified as “yes” if sufficient, “no” if insufficient, and “unclear” if the author does not describe this approach well, its adequacy can be described.

Two researchers (XZ and TG) separately assessed the risk of bias for each included study. “L”, “H” and “U” are used as codes for evaluating the above-mentioned deviation risk. “L” means low risk of deviation, “H” means high risk of deviation, and “U” means unclear risk of deviation. All the researchers resolved their differences through discussion. When necessary, we will contact the study authors to ask for missing information. When conducting sensitivity analysis, high-risk trials should be considered.

### 2.6 Data synthesis and analysis

Review Manager Software (RevMan, Version 5.3 for windows, The Cochrane Collaboration, Oxford, England) has been used to analyze and synthesize the outcomes.Clinical heterogeneity is the primary source of heterogeneity in the systematic reviews of traditional Chinese medicine (TCM). Clinical heterogeneity can be derived from the potential factors such as the manufacturer of Shenfu Injection.^[27]^ Quantitative synthesis has been done when clinical heterogeneity is not considered by at least 2 authors in discussion. Continuous variable has been described by mean difference, *P*–value, and 95% confidence interval. For dichotomous outcomes, the relative risk has been used, with 95% confidence interval and *P*-values, to evaluate the efficacy and safety of Shenfu Injection. *I*^*2*^ test has been used to judge the heterogeneity of meta-analysis. *I*^*2*^ value >50% or more will be considered as an indication of substantial heterogeneity. If heterogeneity exists in the pooled studies, the data have been analyzed using a random effects model. Otherwise, a fixed effect model has been adopted. If there is significant clinical heterogeneity, the cause of heterogeneity should be explored, and sensitivity analysis or subgroup analysis should be performed when necessary. Sensitivity analysis has been used to ensure the robustness of results by eliminating low-quality trials. Subgroup analysis will be performed according to the characteristics of the study subjects, such as different interventions, treatment duration, and outcome measures. If the data extraction is insufficient, qualitative analysis will be adopted.

### 2.7 Publication bias

When the number of studies included in the meta-analysis is not less than 10, use the funnel chart to analyze the publication deviation. If the number of studies included is <10, the Egger test is applied. The analysis software is R3.5.1 for windows.

### 2.8 Quality of evidence

This study evaluates evidence based on grading standards, which are recommended evaluation, development, and evaluation grading standards. These factors that may reduce the quality of evidence should be considered, such as the limitations of study design, inconsistency of results, indirectness of evidence, imprecision, and publication bias. In addition, large-scale effects, confounding factors that may reduce effects, and dose response gradients that improve the quality of evidence cannot be ignored. Grading of Recommendations Assessment, Development, and Evaluation Pro GDT online software will be used to form a summary table of survey results (SoF table).

## 3 Discussion

TCM has been used in China for thousands years, and the effective component injections of TCM have been widely used.^[28]^ Shenfu injection is a preparation of Shenfu Decoction, whose active ingredients include ginsenoside and aconitine.^[29]^ Many studies have shown that Shenfu injection can strengthen the heart, boost blood pressure, resist hypertension, improve hemodynamics, regulate immunity, suppress inflammation and increase hypoxia tolerance.^[30]^ Modern pharmacological studies have shown that ginsenosides can improve the metabolism of ischemic myocardium, scavenge free radicals, protect myocardial ultrastructure, and reduce myocardial load; aconitine can enhance myocardial contractility, improve coronary circulation, and reduce the effects of acute myocardial ischemia.^[31]^ In addition, aconitine contains norepinephrine sasolino, which has an excitatory effect on beta receptors and adrenergic receptors, and can significantly increase cerebral blood flow by improving mean arterial pressure.^[32]^ Moreover, Shenfu injection can block the vicious circle of inflammatory response by reducing the expression of the necrosis factor, improve systemic microcirculation, and prolong the time of hypoxia tolerance.^[33,17]^

In China, although there have been many clinical trial reported the efficacy of Shenfu injection in shock, there are still some problems in these trials, such as small sample size and low methodological quality.^[34]^ Also, Correcting microcirculation disorders plays a vital role in the prognosis of shock patients.^[12]^

Therefore, the purpose of our research is to conduct a meta-analysis to evaluate its clinical efficacy and safety in shock patients, as well as its impact on the microcirculation. We hope that this study can further provide reference for clinical practice.

### 3.1 Ethics and dissemination

This review does not require ethical approval because the included studies are published data and do not involve the patients’ privacy. The results of this review will be reported in accordance with the PRISMA extension statement and disseminated to a peer-reviewed journal.

## Author contributions

**Conceptualization**: Xuemei Zhang.

**Methodology**: Ting Guo, Kaichen Zhang, Wenhui Guo.

**Software**: Xing An.

**Supervision**: Peiyang Gao.

**Writing – original draft**: Xuemei Zhang, Ting Guo, Wenhui Guo.

**Writing – review & editing**: Xuemei Zhang, Ting Guo, Peiyang Gao.
